# Superiority of targeted RNA sequencing for fusion detection and subtype diagnosis in Chinese sarcoma patients: a multicenter study

**DOI:** 10.1186/s40164-025-00663-2

**Published:** 2025-05-14

**Authors:** Gu Jin, Dandan Zhao, Baoming Wang, Xuejiao Liu, Quanyu Yang, Zhengchuang Liu, Qiong Yang, Jianhua Zhu, Jie Zhang, Wei Li, Xiaojuan Wang, Chunyang Wang, Tonghui Ma, Jiayong Liu

**Affiliations:** 1 Zhejiang Cancer Hospital, Institute of Basic Medicine and Cancer (IBMC), Chinese Academy of Sciences, Hangzhou, 310022 Zhejiang China; 2 Genecn-Biotech, Hangzhou, 310022 China; 3 Zhejiang University, Hangzhou, 310058 China; 4 Key Laboratory of Gastroenterology of Zhejiang Province, Zhejiang Provincial People’s Hospital, People’s Hospital of Hangzhou Medical College, Hangzhou, 310014 China; 5 Clinical Research Center, Zhejiang Provincial People’s Hospital, People’s Hospital of Hangzhou Medical College, Hangzhou, 310014 China; 6 General Surgery, Cancer Center, Department of Breast Surgery, Zhejiang Provincial People’s Hospital (Affiliated People’s Hospital, Hangzhou Medical College), Shangtang Road 158, Hangzhou, 310014 China; 7 Zybio Inc, Chongqing, 400000 China; 8 Key Laboratory of Carcinogenesis and Translational Research (Ministry of Education/Beijing), Department of Bone and Soft Tissue Tumor, Peking University Cancer Hospital & Institute, Beijing, 100142 China

**Keywords:** Sarcoma, Targeted RNA sequencing, Fusion, Not otherwise specified, Reclassification

## Abstract

Sarcomas are rare, heterogeneous mesenchymal malignancies with notably high misdiagnosis rates. Despite sarcoma patients in China representing about one-quarter of the global disease burden, large-scale NGS-based diagnostic studies remain scarce, with limited sample sizes failing to capture the extensive subtype complexity of sarcomas. To address diagnostic gaps, we conducted the largest multicenter study in China involving 788 patients with soft tissue or bone sarcomas. All samples underwent targeted RNA sequencing (Fusioncapture) alongside standard histopathology, immunohistochemistry, and DNA-based next-generation sequencing (NGS). Compared with DNA-NGS, RNA-based profiling clarified ambiguous fusion calls and uncovered numerous additional and clinically relevant events, including 281 fusions not captured by the DNA panel. Notably, 114 recurrent alterations were strongly subtype-associated, and 20 newly identified receptor tyrosine kinase fusions had therapeutic significance, expanding targetable cases from 3.3% to 6.5%. Furthermore, integrated RNA data led to subtype reclassification in 11.9% of patients, including 22% of those initially diagnosed as “not otherwise specified”. These findings confirm the utility of targeted RNA sequencing for detecting transcriptionally active fusions, refining pathological classifications, and identifying actionable variants in Chinese sarcoma patients. Despite retrospective design and limited orthogonal validation of some fusions, our results strongly support incorporating RNA-based assays into routine clinical workflows. Ultimately, this integrated approach can improve diagnostic precision, guide personalized treatment strategies, and enhance outcomes for sarcoma patients.

**Supplementary Information:**

The online version contains supplementary material available at 10.1186/s40164-025-00663-2.

**Dear editor**

Sarcomas are a rare and diverse group of mesenchymal malignancies encompassing more than 100 subtypes [1, 2]. Due to significant histological overlap, the misdiagnosis rate for certain soft tissue sarcomas can be as high as 40% [3]. In China, where patients account for approximately one-quarter of the global sarcoma burden, such diagnostic complexities impose substantial clinical and economic challenges.

Conventional molecular tests, including fluorescence in situ hybridization (FISH), immunohistochemistry (IHC), and polymerase chain reaction (PCR), have improved diagnostic precision for select gene fusions, but their limited coverage can miss novel or rare events [4, 5]. Next-generation sequencing (NGS) expands genomic interrogation, though DNA-based panels may overlook intronic breakpoints or non-expressed rearrangements [6–9]. By detecting spliced, functional transcripts, RNA-based NGS offers a broader view of potential oncogenic fusions [8–10].

To date, there is limited data on the clinical impact of targeted RNA sequencing in large cohorts of Chinese sarcoma patients, and previous Chinese sarcoma NGS studies have limited sample sizes, failing to encompass the complexity of sarcoma subtypes, undermining their representativeness [11–13]. To address these gaps, we conducted the largest multicenter investigation to date in China, enrolling 788 patients to systematically assess the performance of combined DNA and RNA NGS in diagnosing sarcomas. This integrated approach enabled us to uncover subtypes with high misdiagnosis rates, refine diagnostic accuracy, and illuminate novel actionable fusions. Our findings aim to guide clinicians in accelerating precise, personalized care for sarcoma patients in China and, by extension, in other populations with similar diagnostic challenges.

In this multicenter retrospective study, we enrolled 788 patients diagnosed with soft tissue or bone sarcomas and performed targeted RNA sequencing (Fusioncapture) on all available tumor samples. Corresponding histopathology and IHC results, as well as DNA-based NGS data, were retrieved for comparison. Fusions detected uniquely by RNA sequencing were subjected to orthogonal validation (e.g., FISH or Sanger sequencing) when additional specimen material was available. Detailed methodology, including panel composition, sequencing protocols, bioinformatics pipelines, validation assays, and inclusion criteria, are provided in the Additional file 1.

Our RNA-based targeted panel demonstrated high concordance with DNA-NGS for shared fusion targets (Fig. 1A). Among overlapping coverage regions, RNA sequencing achieved a sensitivity of 93.5% and a specificity of 98.8% (Additional file 2: Table S1). Notably, RNA sequencing clarified ambiguous DNA-level calls. Of seven DNA-only fusions, four were tested: two (*intergenic*::*NTRK1*, *PLEKHH2*::*ALK*) proved FISH-negative, and two (*PAPPA2*::*NTRK1*, *SYT11*::*NTRK1*) were FISH-positive but IHC-negative, indicating non-functional events (Fig. 1A-E). Conversely, among eight RNA-only fusions evaluated, five samples available were orthogonally validated and six held clear diagnostic or therapeutic relevance (Fig. 1A and F-K; Additional file 2: Table S2). Beyond this, the RNA approach detected 281 additional fusions in 221 patients that were not covered by the DNA panel (Fig. 1L). Among these, 114 were recurrent alterations strongly associated with distinct sarcoma subtypes and thus offered meaningful diagnostic guidance (Fig. 1M). Additionally, RNA sequencing identified 20 receptor tyrosine kinase fusions with potential therapeutic implications, which were previously undetected due to the limited coverage of the DNA panel. When combined with the 5 therapeutically relevant fusions detectable only by RNA sequencing within the shared coverage region, the total number of targetable cases expanded significantly, increasing the proportion from 3.3% (DNA only) to 6.5% (RNA plus DNA) (Fig. 1M; Additional file 2: Table S3). In addition, a significant number of novel fusions were detected in the 788 sarcoma cases; among these, 13 showed potential clinical utility, with three potentially useful for pathological classification and 10 offering therapeutic value (details provided in Additional file 1: Fig. S5).

**Fig. 1:**
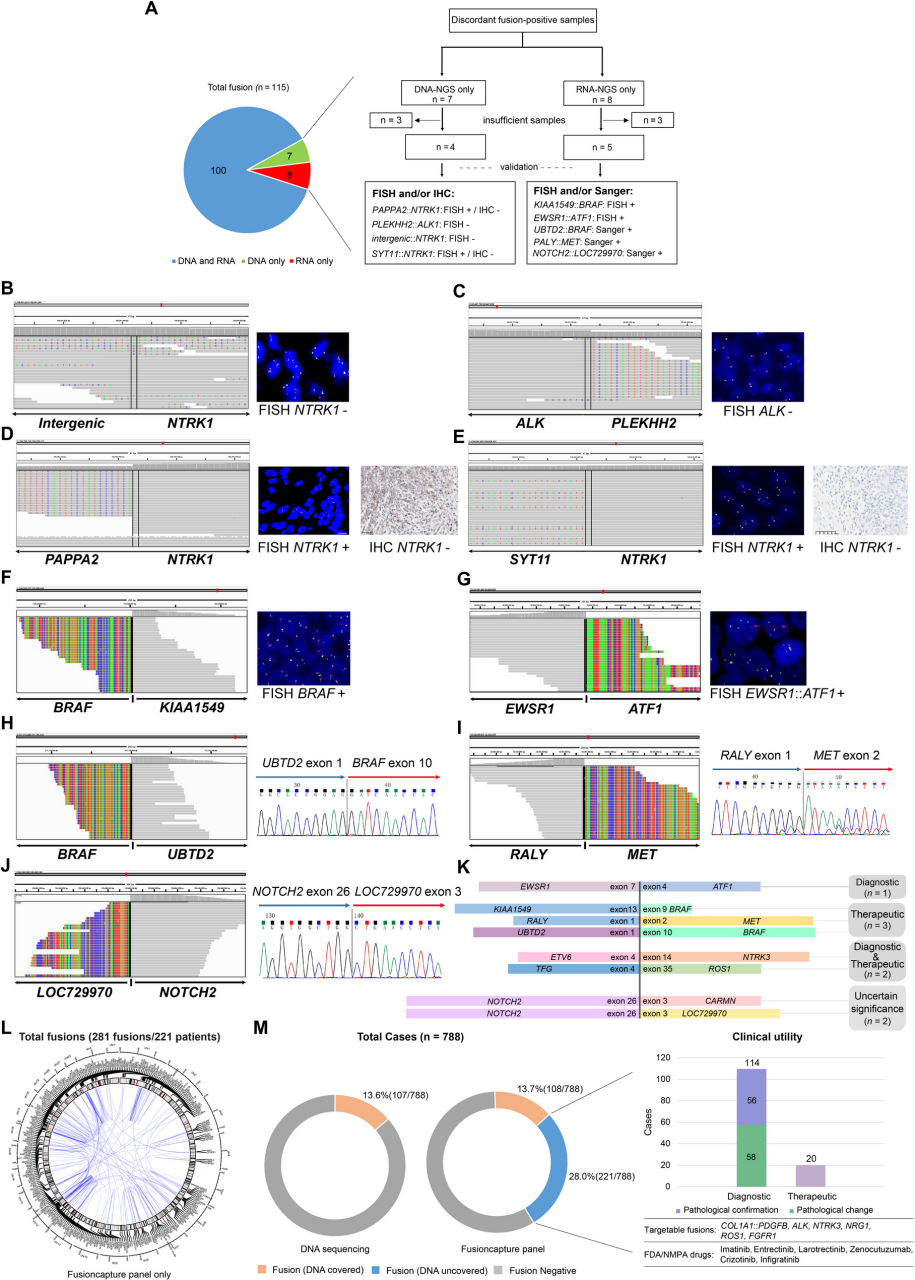
Comprehensive evaluation of RNA-based Fusioncapture versus DNA-NGS in fusion detection and orthogonal validation in sarcoma. (**A**) Representative sarcoma cases showing fusion detection discrepancies between RNA-based Fusioncapture™ and DNA-based Onco PanScan™ panels within the 28 overlapping genes shared by both assays. The figure includes DNA-positive/RNA-negative and RNA-positive/DNA-negative fusion calls, along with their orthogonal validation results (e.g., FISH, Sanger sequencing, IHC). (**B - E**) Verification for 4 DNA-NGS positive/RNA-NGS negative fusions. FISH assay was performed on all the four patients, including *Intergenic*::*NTRK1*, *PLEKHH2*::*ALK*, *PAPPA2*::*NTRK1*, and *SYT11*::*NTRK1*, respectively. The *Intergenic*::*NTRK1* (**B**) and *PLEKHH2*::*ALK* (**C**) fusions were negative. The *PAPPA2*::*NTRK1* (**D**) and *SYT11*::*NTRK1* (**E**) fusions were considered positive by FISH, but negative by IHC. (**F - J**) Validation of the 5 DNA-NGS negative/RNA-NGS positive fusions. FISH assay was performed on the two other patients, including *KIAA1549*::*BRAF* (**F**), and *EWSR1*::*ATF1* (**G**) and the two fusions were all confirmed as true positive. Sanger sequencing was performed on the three of the 5 patients, as *UBTD2*::*BRAF* (**H**), *NOTCH2*::*LOC729970* (**I**) and *NOTCH2*::*CARMN* (**J**) and all fusions were positive. (**K**) Clinical value of the 8 RNA-NGS detected only fusions. (**L - M**) The landscape of fusions covered only by Fusioncapture panel in the 788 sarcoma patients. (**L**) Circos plot of the 281 gene fusions from 221 cases in all the STS identified by Fusioncapture panel only. (**M**) Increased fusion detection ratio and clinical utility of 221 patients

**Fig. 2:**
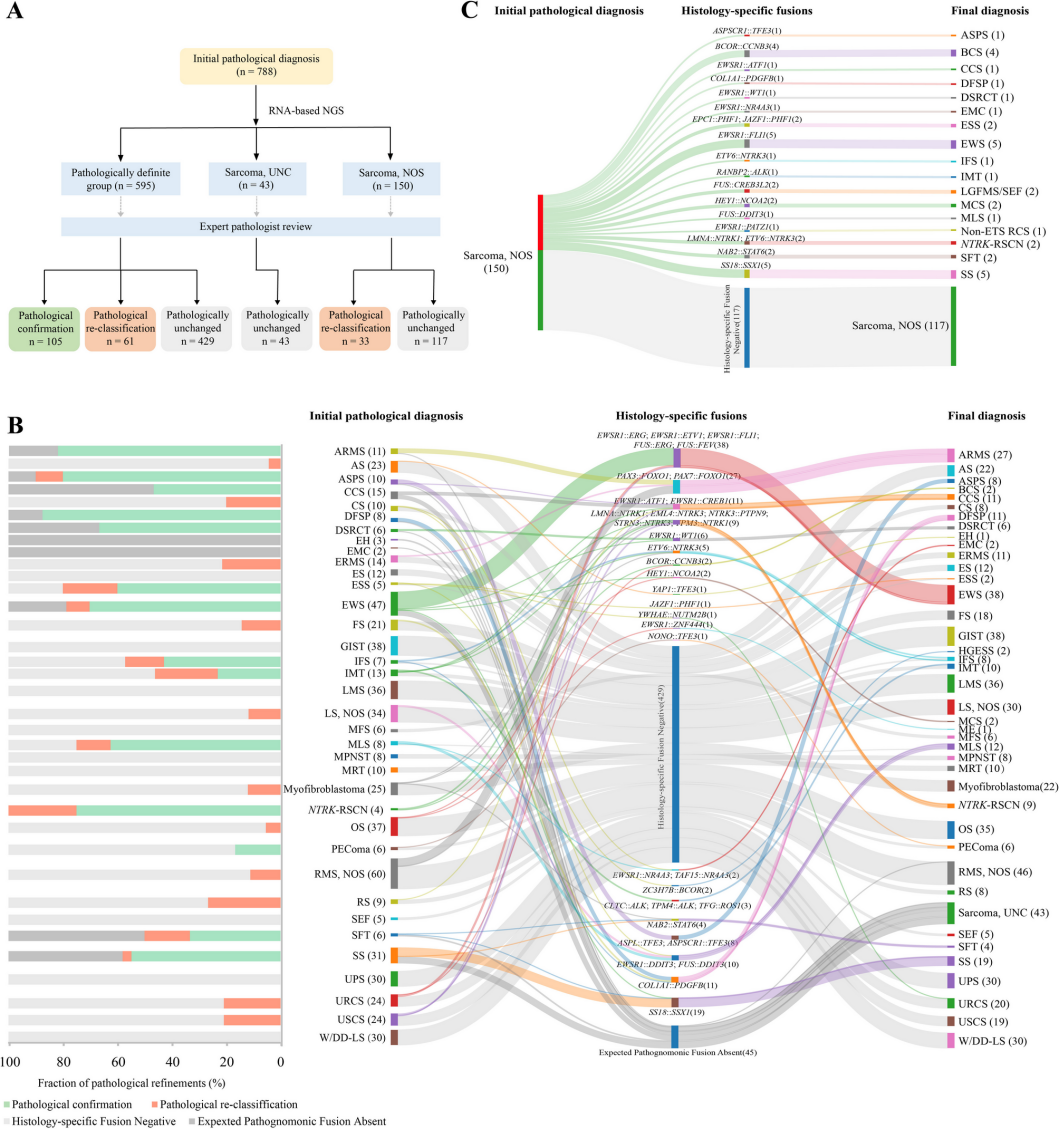
Pathological refinement in 788 STS samples via integrated histology and molecular genetics illustrated schematically. (**A**) Flow chart of corrections of initial pathological diagnosis results in the sarcoma cohort. (**B**) and (**C**) Sankey diagram illustrating the diagnostic changes in pathological definite group of 595 samples and unclassified sarcomas group of 43 samples (**B**) and not specified group of 150 samples (**C**). Bar chart, proportion of pathological refinements for each sarcoma subtype after initial diagnosis; Sankey diagrams: Left nodes, number of cases identified for each subtype according to initial submitted pathology results; Middle nodes, the specific forms and number of histology-specific fusions; right nodes, number of corrections or refinements for each histology as determined by the presence or absence of pathognomonic genomic rearrangements or signatures

Integrating RNA-based molecular data with histopathology significantly impacted patient classification. Overall, 11.9%(94/788) of patients required pathological subtype alterations and 13.3%(105/788) had diagnoses confirmed or refined by incorporating the RNA results (Fig. 2A; Additional file 2: Table S4). Among patients with a defined subtype, 10.3%(61/595) were reclassified after identifying pathognomonic fusions (Fig. 2A, B; Additional file 2: Table S4). Among those initially diagnosed as not otherwise specified (NOS), 22% were reassigned to a specific subtype (Fig. 2C; Additional file 2: Table S4), a higher reclassification rate than previously reported in Western cohorts [10]. This is clinically critical, as accurate subtype diagnosis guides appropriate treatment strategies and eligibility for targeted therapies.

Notably, Fusioncapture performed well even in samples with poor RNA quality, allowing approximately 40% of heavily degraded specimens to be successfully profiled (Additional file 1: Fig. S3B). This enhances its practicality in routine clinical practice, where formalin-fixed, paraffin-embedded tissues can be challenging. Additionally, 23 FISH-prevalidated sarcoma samples were analyzed by RNA-seq to compare fusion detection performance, as detailed in the Supplementary Materials (Additional file 1 and Table S8).

Our study performed on the largest Chinese sarcoma cohort with a broad RNA fusion panel-Fusioncapture, underscores the improved accuracy and utility of RNA-based profiling for both established and novel gene fusions. By providing direct transcriptional evidence, targeted RNA sequencing differentiates true oncogenic drivers from incidental DNA-level events and expands the spectrum of actionable alterations. While our study was retrospective and not all fusions underwent orthogonal validation, the observed diagnostic and therapeutic implications strongly support broader adoption of RNA-based assays in routine clinical practice of sarcoma diagnostics.

## Electronic supplementary material

Below is the link to the electronic supplementary material.

Supplementary Material 1

Supplementary Material 2

## Data Availability

All data needed to evaluate the conclusions in the paper are present in the paper and/or the Supplementary Data. Some datasets generated during and/or analyzed during the current study are not publicly available due to restrictions on participant privacy and content but are available from the corresponding author on reasonable request.
