# Supplementary material for: Superiority of targeted RNA sequencing for fusion detection and subtype diagnosis in Chinese sarcoma patients: a multicenter study
Source: Exp Hematol Oncol. 2025 May 14;14:70. doi: 10.1186/s40164-025-00663-2 (PMC12076812; doi:10.1186/s40164-025-00663-2)
Supplement: Supplementary file 1 — Supplementary Material 1 [file 40164_2025_663_MOESM1_ESM.docx]

**Supplementary Materials and Methods**

**Patients and samples**

In the period of November 2011 to September 2019, 788 patients diagnosed with soft tissue or bone sarcoma from multi-centers (Zhejiang Cancer Hospital, Zhejiang Provincial People's Hospital, and Peking University Cancer Hospital) in China were retrospectively enrolled, and their medical data collected, and the clinical information of these patients were listed in **Table S5**. Biopsies and surgical resections were used to collect tumor tissue samples from patients. This study has been approved by the Ethics Committee of Zhejiang Cancer Hospital (IRB-2021-402), and individual consent for this retrospective analysis was waived; patients below 18 years old were provided with signed informed consent by their legal parents/legal guardians for the research use of clinical data and publication.

**DNA Sequencing and Data Analysis**

Genomic DNA was isolated from FFPE samples or frozen tissues and analyzed using a capture-based next generation sequencing panel targeting 825 cancer-related genes (Onco PanScan™️, Genetron Health, Beijing, China), as described previously (1). Trimmomatic (v0.36) was used for quality control to rule out adapters and poor-quality regions from the raw sequencing data. Sequence data were mapped to the reference human genome (hg19) using the Burrows–Wheeler Aligner tool (BWA, v0.7.10). Structural variations (SVs) were determined using GeneFuse version v0.6.1 (https://github.com/OpenGene/GeneFuse). Following the Exome Aggregation Consortium's rules, variations from 1000 Genomes and dbSNP having a population frequency of more than 0.1% were eliminated (ExAC). Fusions will be reported if the variant allele frequency (VAF) ≥ 1% (hotspot mutations) or ≥ 5% (non-hotspot mutations) and four or more supporting reads that included both split reads and span reads were found in the tumor samples.

**Targeted RNA Sequencing and Data Analysis**

Total RNA was isolated from tumor samples, including both FFPE and frozen tissues, using the RNeasy FFPE Kit (Qiagen, Hilden, Germany) and subjected to Fusioncapture™ (Genetron Health, Beijing, China), which targeted the full transcripts of 395 cancer-related genes **(Additional file 2: Table S6)** as described previously (2). Briefly, 200ng purified total RNA was first converted to double-stranded cDNA through reverse transcription reaction. To prepare genomic libraries, the double-stranded cDNA was fragmented and constructed. The follow-up hybridization-captured libraries were sequenced on the Illumina NovaSeq 6000 platform (Illumina, San Diego, USA) with 150 base pair (bp) paired-end reads. On average, approximately no less than 30 million reads were generated per sample. The sequencing reads were mapped to UCSC hg19 through HISAT2-2.0.5, and FusionMap software was used to identify gene fusions. Fusions were called with at least four unique pairs of supporting reads spanned over the breakpoints between the two partners.

**RNA-Seq**

Total RNA was extracted from FFPE tumor samples using the RNeasy FFPE Kit (Qiagen, Hilden, Germany). The quantity and quality of RNA was then determined by the Qubit RNA HS Assay Kit (Thermo Fisher Scientific, Waltham, MA) and DV200 (the percentage of RNA fragments longer than 200 nucleotides) RNA Nano assays with the Agilent 2100 Bioanalyzer System (Agilent Technologies, Santa Clara, CA). Followed by the rRNA depletion, cDNA synthesis, and NGS library preparation using the KAPA RNA HyperPrep Kit with RiboErase (Kapa Biosystems, Wilmington, MA) according to the manufacturer’s protocol. The RNA libraries were sequenced on an Illumina HiSeq genome analyzer with 150 base pair (bp) paired-end reads (Illumina; San Diego, CA, USA). Each sample yielded an average of no less than 150 million reads. Raw RNA-seq data were de-multiplexed, aligned to the reference genome (hg19, UCSC), and quality-filtered. Then fusion was called by STAR-Fusion version 1.5.0 (<https://github.com/STAR-Fusion/STAR-Fusion/wiki>). Fusions were considered positive if they met the criteria of supporting reads ≥ 20 (including both split and span reads) and frequency ≥ 0.01, while those with fewer than 4 supporting reads were filtered out. All the fusions identified were further verified by manual inspection using the Integrative Genomics Viewer (IGV).

**Fluorescence in Situ Hybridization (FISH), RT-PCR and Sanger Sequencing**

To validate the fusions identified by our Fusioncapture panel but not by the DNA assay and the accuracy of Fusioncapture panel, Sanger sequencing and FISH were performed. For Sanger sequencing, cDNA was prepared from 2.5 μg of total RNA using SuperScript™ VILO™ MasterMix (11755-250, invitrogen) reaction conditions and the DEPC-treated water was used as negative RT control. PCR was performed with fusion specific primers. Reference sequence of fusion gene transcripts can be found at National Center of Biotechnology Information Reference Sequence Database. PCR products were separated on 1.5% to 3.0% agarose gels, purified with the QIA quick Gel Extraction kit (Qiagen) and sent for sequencing at Beijing Genomics Institute, China. Fusion sequences were then aligned with the sequence detected by the Fusioncapture panel to verify the accuracy. FISH was performed on interphase nuclei on 5μm FFPE tissue sections using specific FISH probes. A total of 200 nuclei per probe within matched hematoxylin and eosin marked tumor areas were visually evaluated with fluorescence microscopy. Break-apart FISH probes and Dual Fusion FISH probes were used for validation of *SS18*, *EWSR1*, *NTRK1*, *ALK*, *BRAF*, *FOXO1*, *RET*, *COL1A1*::*PDGF*B, and other relevant fusion partners, and these probes were purchased from ZytoVision (Bremerhaven, Germany) unless otherwise noted. For immunohistochemistry (IHC) validation, the pan-Trk antibody (clone EPR17341, Abcam, UK; catalog number ab181560) was used for the detection of *NTRK1* fusions in relevant cases.

**Comparative Analysis of Fusion Detection**

To evaluate the performance of the RNA-based Fusioncapture™ panel for fusion detection, comparative analysis was performed using results from the DNA-based Onco PanScan™ panel as the reference standard. Since the two panels differ in gene coverage (825 genes in the DNA panel and 395 in the RNA panel), only fusion detected genes present in both panels (n = 28) were included in the comparative analysis to ensure an appropriate match of detectable targets. Specifically, the analysis focused on structural variants (SVs) identified in both panels, as indicated by the “*” symbols in the Gene columns of Table S6 and Table S7 in Additional file 2. A total of 28 genes were identified as common fusion genes detected by both panels, and these genes served as the basis for evaluating fusion detection. Sensitivity and specificity were calculated based on fusion events detected by the DNA panel within this shared subset of genes. Fusion events were considered true positives when detected by both assays, false positives when detected only by DNA sequencing, and false negatives when detected only by RNA sequencing. Orthogonal validation (FISH or Sanger sequencing) was performed on selected discordant cases when material was available.

**Supplementary Results**

**The Detection Performance of targeted RNA sequencing based Fuisoncapture in Fusion Detection**

RNA reference material Seraseq^®^ FFPE Tumor Fusion RNA v4 was purchased and used to evaluate the performance of Fusioncapture panel at different sizes of sequencing data (10, 8, 6, 4 and 2 Gigabases (G)). Even with 2 G of sequencing data, all 17 panel-covered fusions were accurately detected, and the minimum supported reads were 14 for *SLC34A2*::*ROS1*. The number of supporting reads was linearly correlated with the sequencing sizes **(Additional file 1: Fig. S1A)**, and the limit of detection (LOD) for the panel can be as low as 2 copies per nanogram of total RNA input **(Additional file 1: Fig. S1B)**. Further, H2228 (*ALK*::*PTPN3* and *EML4*::*ALK*) and Reh (*ETV6*::*RUNX1* and *RUNX1*::*PRDM7*) cell lines were mixed with different proportions to investigate the impact of tumor cell contents on the performance of fusions detection. As a result, all of the four fusions could be detected at a low proportion of less than 10% **(Additional file 1: Fig. S1C)**. Moreover, an excellent linear correlation between supporting reads and the percentage of cell mixture was also observed in these fusions **(Additional file 1: Fig. S2)**. Beyond that, the panel also demonstrated high repeatability and reproducibility, with all fusions detected and CV (Coefficient of Variation, the ratio of the standard deviation to the mean, expressed as a percentage, representing the variation in fusion mutation detection frequency across replicates) values of fusion-supporting reads < 20% in the triplicate of three clinical sarcoma samples harboring *COL1A1*::*PDGFB*, *PAX3*::*FOXO1*, and *SS18::SSX1,* respectively **(Additional file 1: Fig. S1D, S1E)**.

**Validation of Fusion Gene Detection in Clinical Samples**

To further verify the accuracy of Fusioncapture in clinical samples, 23 sarcoma Formalin-fixed paraffin-embedded (FFPE) samples with known rearrangements identified by FISH were also validated using RNA-seq **(Additional file 2: Table S8)**. Among the 23 clinical samples, 14 were confirmed as FISH fusion-positive. Compared with Fusioncapture, which detected all the 14 positive fusions in these samples, 5 of these 14 fusions, including 4 *SS18*::*SSX1* and 1 *SS18*::*SSX9* fusions, were determined to be negative by RNA-seq because of the low number of supporting reads **(Additional file 1: Fig. S3A; Additional file 2: Table S8)**. Additionally, 5 fusions were found in the screen of Fusioncapture in these samples, and 3 out of these fusions including *ATIC*::*XRCC5*, *ELN*::*MAGI2* and *RPTOR*::*CLTCL1* were also detected by RNA-Seq, while the other 2 fusions, *HMGA2*::*COL1A2* and *RAF1*::*PRKCI*, were left out **(Additional file 1: Fig. S3A; Additional file 2: Table S8)**. Moreover, the median abundance of fusion-supporting reads of Fusioncapture was significantly higher than that of RNA-seq (1947.8 vs. 2.1, P < 0.0005, **Additional file 1: Fig. S4**), implying that Fusioncapture may have obvious advantages for fusion detection in sarcoma samples with low tumor purity than RNA-seq. In addition, 106 sarcoma FFPE samples with DV200 values below 30%, which suggested to be too degraded for RNA-Seq, were involved to evaluate the performance of Fusioncapture in low-quality RNA. Finally, 40% (42/106) of these poor-quality samples met quality control and were successfully sequenced **(Additional file 1: Fig. S3B; Additional file 2: Table S9)**.

**Novel Fusions Detection with** **targeted RNA sequencing**

A significant number of novel fusions were detected in the 788 sarcoma cases. Of the identified novel fusions, 13 showed potential clinical utility, with three fusions potentially useful for pathological classification and 10 fusions with potential therapeutic value **(Additional file 1: Fig. S5A)**. For instance, the *EWSR1*::*COLCA2* and *MAMLD1*::*SSX1* fusion showed promise in aiding the diagnosis of Synovial sarcoma (SS) **(Additional file 1: Fig. S5B, C)**. Additionally, tumors with the novel fusion *SPPL2A*::*BRAF* and *NTRK3*::*PTPN9* exhibited the potential to benefit from TKIs **(Additional file 1: Fig. S5D, E)**. To confirm the presence of these novel fusions, specimens with available material underwent cross-validation through Sanger sequencing or FISH (**Additional file 1: Fig. S5B-E**). The additional confirmation further supported the significance of these findings and their potential application in the clinic. Overall, RNA-based Fusioncapture assay provides valuable insights into the molecular landscape of sarcomas and identifies novel fusions with potential clinical utility, which have the potential to improve the accuracy of diagnosis and facilitate more targeted and effective treatments for patients with sarcomas.

**Supplementary Discussion**

Owing to the splicing out of introns to allow more region to be covered and providing direct evidence of fusion transcripts, RNA-based sequencing was considered as a more optimal way to detect fusion than DNA-based sequencing (3). However, comparing on the performance between DNA-NGS and RNA-NGS for fusion detection was difficult because of significant differences in the strategy of fusion detection. Peng et al found that only 10 of the 12 positive fusions discovered by DNA-NGS were recognized at transcriptional level, which seems that RNA-NGS showed inferior sensitivity than DNA-NGS in fusion detection (4). Moreover, the limited number of fusion positive patients in the cohort has the potential to introduce bias into the performance of fusion detection because only a small number of fusion types involved. Nevertheless, Solomon et al discovered that the detection rate of *NTRK* fusion by RNA-NGS was significantly higher than that by DNA-NGS (5). These conflicting results suggest that further larger confirmative studies involving more diverse fusion types are needed to compare the performance between DNA-NGS and RNA-NGS for fusion gene detection. In this study, 788 sarcomas, the largest cohort of Chinese sarcoma patients, which contains the most diverse type of fusions in solid tumor were analyzed.

Within the detection range covered by DNA and RNA panels, RNA-NGS exhibits excellent concordance of fusion detection with DNA-NGS, with a sensitivity of 93.5% and a specificity of 98.8%. In terms of specificity, RNA-NGS verified 7 false positive samples from the DNA-NGS result, including 3 *NTRK1* fusions (Fig. 1A-E; Additional file 2: Table S2). Solomon et al reported that 43% (46/107) of the *NTRK* fusions at DNA level did not exhibit a transcribed *NTRK* fusion transcript when reflexed to RNA testing (5). While these missed fusions weren’t further confirmed by other methods, it is possible that these cases called false positive by DNA-based sequencing could actually be a false negative by RNA sequencing. In this work, *PAPPA2*::*NTRK1 and SYT11*::*NTRK1* identified by DNA-NGS was further validated by FISH and IHC, and presented *NTRK1* FISH positive but pan-Trk negative result, which suggest RNA-based sequencing can provide direct information to help identify functional fusions at DNA level. More interestingly, 1 case with *Intergenic*::*NTRK1* fusion at DNA level, however, FISH negative was obtained by the orthogonal validation. The reasons for the conflicting result between DNA-NGS and FISH may attribute to the rearrangement was wrongly captured during library preparation or sequencing (6). Otherwise, the insufficient splitting of the signals by FISH may also be responsible (7). In contrast, RNA-NGS utility can circumvent these issues, making it a more reliable approach for fusion detection in sarcoma.

In terms of sensitivity, the RNA-based Fusioncapture panel found an additional 8 cases deemed negative by DNA-NGS, most of which were confirmed to be true by a third method. The high false-negative results of DNA NGS assays may mainly because that most genomic breakpoints that produce fusion genes occur in introns, which cannot always be fully covered by hybrid capture–based NGS because they include diverse sequences (8). Take *ROS1* fusion for example, which serves an important diagnostic and/or therapeutic fusion abnormalities in IMT, owing to the numerous repetitive elements enriched in the intron sequence, *ROS1* fusions were frequently missed by DNA NGS assays (3, 9). Although our study was not designed to evaluate the sensitivity for *ROS1* detection, our findings are consistent with false-negative rates from prior studies in NSCLC (1/3 vs 4/14 or 10/33) and highlight the potential pitfalls of *ROS1* fusion detection when relying exclusively on DNA-level events across different cancer types (3, 10). In a different way, *BRAF* fusions whose involved intron were well covered but not detected by DNA-NGS in our study may contribute to the low tumor purity, as no other mutations, including silent ones, were detected in both samples. Hence, our findings highlight the utility of RNA sequencing for confirming active transcription and reducing missed structure variants from DNA sequencing.

Besides the superior ability in detecting fusions covered by both assays, 281 fusions from 221 cases were only identified by RNA sequencing due to the missing coverage of the DNA panel. Due to assay design limitations, it is impractical to tiling hotspot introns or partners to achieve comprehensive detection of driver gene fusions by DNA sequencing. Therefore, it is conceivable that DNA-based sequencing approaches may fail to reliably detect certain significant gene fusions that occurred in rare introns. For example, different from the hot breakpoints involving intron 19 and 20 of *ALK* fusion, introns 31-35 of *ROS1* fusion, and intron 11 of *NTRK3* fusion in pan-cancer, the fusion positive sarcomas with rare introns of *ALK* (intron 3), *ROS1* (intron 1) and *NTKR3* (intron 3) were only detected by RNA-NGS. In addition, long genomic introns can also interfere with gene fusion detection. For example, intron 13 of *NTRK3* is a well-known technically challenging domain, which, due to its length, cannot be fully covered by targeted DNA-based NGS assays, leading to frequent missed detections of *NTRK3* fusions (7).

### In comparison to previous studies, our findings align with the growing body of evidence supporting the utility of RNA-NGS in fusion gene detection. In our study, RNA-based Fusioncapture demonstrated high sensitivity (93.5%) and specificity (98.8%) for fusion gene detection in sarcoma samples. These values indicate that RNA-NGS is a reliable tool for fusion detection, with minimal false positives and a high detection rate for clinically relevant fusions. Previous studies further validate the advantages of RNA-NGS. For example, Solomon et al. showed that RNA-NGS outperformed DNA-NGS in detecting *NTRK* fusions, with RNA-NGS exhibiting higher sensitivity (7). Additionally, studies by Ji et al. and Erin E. Heyer et al. highlighted the enhanced sensitivity of RNA-NGS. Ji et al. reported a sensitivity and specificity of 100% in a limited sample set (11), while Erin E. Heyer et al. demonstrated that RNA sequencing increased the fusion diagnostic rate from 63% to 76% (12). Moreover, RNA-NGS offers functional evidence of gene fusions, addressing the limitations of DNA-NGS in detecting intronic breakpoints or non-expressed rearrangements. This was demonstrated in our study, where RNA-NGS identified 281 fusions that were missed by DNA-NGS. This ability to detect a broader range of fusion events underlines the potential of RNA-NGS to improve clinical outcomes by uncovering clinically significant alterations that might otherwise be overlooked.

Although RNA sequencing is increasingly recognized as a valuable tool in sarcoma diagnostics (12, 13) , most prior studies have focused on specific subtypes or limited cohorts. While routine use of RNA-based panels—including commercial assays such as the Archer FusionPlex Sarcoma panel—has demonstrated substantial diagnostic utility, large-scale comprehensive evaluations remain relatively limited. Demicco et al reported 20 patients (10%, 20/206) who were re-classified from the initial diagnosis to a different type of sarcoma, based on the histologic appearance and molecular feature (14). However, only a few histological types were examined (dedifferentiated liposarcoma, leiomyosarcoma, undifferentiated pleomorphic sarcoma, myxofibrosarcoma, malignant peripheral nerve sheath tumor, and synovial sarcoma). Zhang et al. included nearly 30 subtypes of sarcoma and 14.8% (13/88) patients ultimately modified their preliminary histology-based classification (15). However, the narrow gene set in the RNA-NGS panel (67 genes) will surely leave out some fusions, which may be crucial to the carcinogenesis of sarcoma. Additionally, prior studies found that 30–50% of Chinese sarcomas were represented by unclassified samples (16, 17). However, only 3 unclassified samples were included in the Zhang et al. study. In our investigation, the RNA-NGS data were integrated with histological diagnosis to reassess the largest cohort of Chinese sarcoma patients with various WHO histological classifications. The results exhibited that 11.9% (94/788) of patients required reclassification. In the definite subtype group, the subtypes were altered in 10.3% (61/595) of patients. Such a high reclassification proportion may be due to the complexity of the sarcoma histology. More remarkably, besides those samples with classified subtypes, up to 22% (33/150) of patients in the group of samples with ambiguous subtypes could be refined to a certain sarcoma subtype characterized by recurrent genetic aberrations. Additionally, the utilizing of RNA-NGS not only functionally discriminates for potential therapy kinase fusions detected at the DNA-NGS level, but also nearly doubled the scope of potential druggable sarcoma patients (3.3% to 6.5%). These findings illustrated the high impact of molecular markers on future sarcoma classification and treatment, and the targeted RNA sequencing may become a routine screening tool for sarcoma diagnosis and targeted therapies in the future through timely and reliable molecular profiling.

In addition to confirming transcriptional activity and expanding fusion detection beyond DNA-based methods, our identification of novel fusions, such as *EWSR1*::*COLCA2* and *MAMLD1*::*SSX1*, provided subtype-defining evidence for synovial sarcoma (SS). These fusions were detected in histologically confirmed cases, and validated via orthogonal methods (Sanger sequencing or FISH), confirming their authenticity and relevance. The presence of these fusions enabled the reclassification of these cases as SS, suggesting they may represent atypical but subtype-specific molecular markers of SS. While *EWSR1*::*SSX1* is the primary genetic driver in SS, previous studies have identified alternative fusion partners involving *EWSR1* or *SSX1*, indicating the molecular diversity of SS (18). These alternative fusions may represent distinct SS subtypes, thus expanding the genomic landscape of the disease (19). This may expand the genomic landscape of SS and highlights the clinical utility of comprehensive RNA-based fusion profiling in challenging diagnostic settings. However, further validation in larger cohorts is needed to assess the clinical significance of these findings.

In spite of its novelty, the present study has several limitations. One of the limitations was that out of the 8 cases detected only by RNA-NGS within the co-coverage of DNA and RNA panel, only samples with enough specimens were further verified. It was possible that these non-validated samples, referred to as false negatives at the DNA level, might actually be false positives by RNA sequencing. In addition, the comparison between targeted RNA sequencing and RNA-seq would be more adequate if RNA-seq detection was performed in these samples with DV200 < 30%. However, due to the sample quality/quantity, these samples were not validated by RNA-seq. In addition, more than 100 distinct subtypes were identified in sarcoma, though we had conducted the largest sarcoma cohort in the Chinese population so far, there are still some rare subtypes that were not covered. Besides, there were some rare subtypes of sarcoma with a limited number of enrolled patients, which may affect the proportion of molecular-guided reclassification in our cohort. However, these rare subtypes only accounted for a small proportion of sarcoma patients, and they didn’t cause significantly bias in our conclusions.

**Supplementary Abbreviations**

ARMS, Alveolar Rhabdomyosarcoma; BCS, Sarcoma with BCOR genetic alterations; AS, Angiosarcoma; ASPS, Alveolar soft part sarcoma; CCS, Clear cell sarcoma; CS, Chondrosarcoma; DFSP, Dermatofibrosarcoma protuberans; DSRCT, Desmoplastic small round cell tumor; EH, Epithelioid hemangioendothelioma; EMC, Extraskeletal Myxoid chondrosarcoma; ERMS, Embryonal Rhabdomyosarcoma; ES, Epithelioid sarcoma; ESS, Endometrial stromal sarcoma; EWS, Ewing's sarcoma; FFPE: formalin-fixed paraffin-embedded; FISH: Fluorescence in Situ Hybridization; FS, Fibrosarcoma; GIST, Gastrointestinal Stromal Tumor; HGESS, High grade endometrial stromal sarcoma; IFS, Infantile fibrosarcoma; IHC: immunohistochemical staining; IMT, Inflammatory myofibroblastic tumor; LGFMS/SEF, Low-Grade Fibromyxoidsarcoma/sclerosing epithelioid fibrosarcoma; LMS, Leiomyosarcoma; LOD: limit of detection; LS, Lipoblastomatosis; MCS, Mesenchymal chondrosarcoma; ME, Myoepithelial tumors; MFS, Myxofibrosarcoma; MLS, Myxoid liposarcoma; MPNST, Malignant peripheral nerve sheath tumor; MRT, Malignant Rhabdoid Tumor; Non-ETS RCS, Round cell sarcoma with EWSR1-non-ETS fusions; NGS: Next generation sequencing; NOS, not otherwise specified; NPV: negative predictive value; *NTRK*-RSCN, NTRK-rearranged spindle cell neoplasm; OS, Osteosarcoma; PEComa, Perivascular epithelioid cell tumor; PPV: positive predictive value; RMS, Rhabdomyosarcoma; RS, Rare sarcoma; Sarcoma, UNC, Sarcoma, unclassified; SEF, Sclerosing epithelioid fibrosarcoma; SFT, Solitary fibrous tumor; SS, Synovial sarcoma; UPS, Undifferentiated pleomorphic sarcoma; URCS, Undifferentiated Round Cell Sarcoma; W/DD-LS, Well-differentiated liposarcoma.

UNC (Unclassified Sarcoma): "UNC" refers to sarcomas that initially present with a defined histopathological subtype and are expected to harbor specific pathognomonic gene fusions. However, these characteristic fusions are not detected, and the lack of such molecular features precludes definitive subtype confirmation. For example, solitary fibrous tumors are typically expected to carry *NAB2*::*STAT6* fusions; if such fusions are absent, the sarcoma may be classified as UNC due to the inconsistency between morphology and molecular findings.

NOS (Not Otherwise Specified): "NOS" refers to sarcomas where, even after pathological evaluation, no definitive histological subtype can be assigned.

**References:**

1. Shi M, Wang W, Zhang J, Li B, Lv D, Wang D, et al. Identification of RET fusions in a Chinese multicancer retrospective analysis by next-generation sequencing. Cancer science. 2022;113(1):308-18.

2. Li Y, Wang B, Wang C, Zhao D, Liu Z, Niu Y, et al. Genomic and Transcriptional Profiling of Chinese Melanoma Patients Enhanced Potentially Druggable Targets: A Multicenter Study. Cancers. 2022;15(1).

3. Benayed R, Offin M, Mullaney K, Sukhadia P, Rios K, Desmeules P, et al. High Yield of RNA Sequencing for Targetable Kinase Fusions in Lung Adenocarcinomas with No Mitogenic Driver Alteration Detected by DNA Sequencing and Low Tumor Mutation Burden. Clinical cancer research : an official journal of the American Association for Cancer Research. 2019;25(15):4712-22.

4. Peng H, Huang R, Wang K, Wang C, Li B, Guo Y, et al. Development and Validation of an RNA Sequencing Assay for Gene Fusion Detection in Formalin-Fixed, Paraffin-Embedded Tumors. The Journal of molecular diagnostics : JMD. 2021;23(2):223-33.

5. Solomon JP, Linkov I, Rosado A, Mullaney K, Rosen EY, Frosina D, et al. NTRK fusion detection across multiple assays and 33,997 cases: diagnostic implications and pitfalls. Modern pathology : an official journal of the United States and Canadian Academy of Pathology, Inc. 2020;33(1):38-46.

6. Harismendy O, Ng PC, Strausberg RL, Wang X, Stockwell TB, Beeson KY, et al. Evaluation of next generation sequencing platforms for population targeted sequencing studies. Genome biology. 2009;10(3):R32.

7. Solomon JP, Benayed R, Hechtman JF, Ladanyi M. Identifying patients with NTRK fusion cancer. Annals of oncology : official journal of the European Society for Medical Oncology. 2019;30(Suppl_8):viii16-viii22.

8. Alkan C, Coe BP, Eichler EE. Genome structural variation discovery and genotyping. Nature reviews Genetics. 2011;12(5):363-76.

9. Treangen TJ, Salzberg SL. Repetitive DNA and next-generation sequencing: computational challenges and solutions. Nature reviews Genetics. 2011;13(1):36-46.

10. Li W, Guo L, Liu Y, Dong L, Yang L, Chen L, et al. Potential Unreliability of Uncommon ALK, ROS1, and RET Genomic Breakpoints in Predicting the Efficacy of Targeted Therapy in NSCLC. Journal of thoracic oncology : official publication of the International Association for the Study of Lung Cancer. 2021;16(3):404-18.

11. Ji G, Yao Q, Ren M, Bai Q, Zhu X, Zhou X. An accurate DNA and RNA based targeted sequencing assay for clinical detection of gene fusions in solid tumors. Sci Rep. 2025;15(1):7223.

12. Heyer EE, Deveson IW, Wooi D, Selinger CI, Lyons RJ, Hayes VM, et al. Diagnosis of fusion genes using targeted RNA sequencing. Nat Commun. 2019;10(1):1388.

13. Mercer TR, Gerhardt DJ, Dinger ME, Crawford J, Trapnell C, Jeddeloh JA, et al. Targeted RNA sequencing reveals the deep complexity of the human transcriptome. Nature biotechnology. 2011;30(1):99-104.

14. Comprehensive and Integrated Genomic Characterization of Adult Soft Tissue Sarcomas. Cell. 2017;171(4):950-65.e28.

15. Hu W, Yuan L, Zhang X, Ni Y, Hong D, Wang Z, et al. Development and validation of an RNA sequencing panel for gene fusions in soft tissue sarcoma. Cancer science. 2022;113(5):1843-54.

16. Gan M, Zhang C, Qiu L, Wang Y, Bao H, Yu R, et al. Molecular landscape and therapeutic alterations in Asian soft-tissue sarcoma patients. Cancer medicine. 2022.

17. Amadeo B, Penel N, Coindre JM, Ray-Coquard I, Ligier K, Delafosse P, et al. Incidence and time trends of sarcoma (2000-2013): results from the French network of cancer registries (FRANCIM). BMC cancer. 2020;20(1):190.

18. Yoshida A, Arai Y, Satomi K, Kubo T, Ryo E, Matsushita Y, et al. Identification of novel SSX1 fusions in synovial sarcoma. Mod Pathol. 2022;35(2):228-39.

19. Walker V, Jin DX, Millis SZ, Nasri E, Corao-Uribe DA, Tan AC, et al. Gene partners of the EWSR1 fusion may represent molecularly distinct entities. Transl Oncol. 2023;38:101795.

**Supplementary Figures**


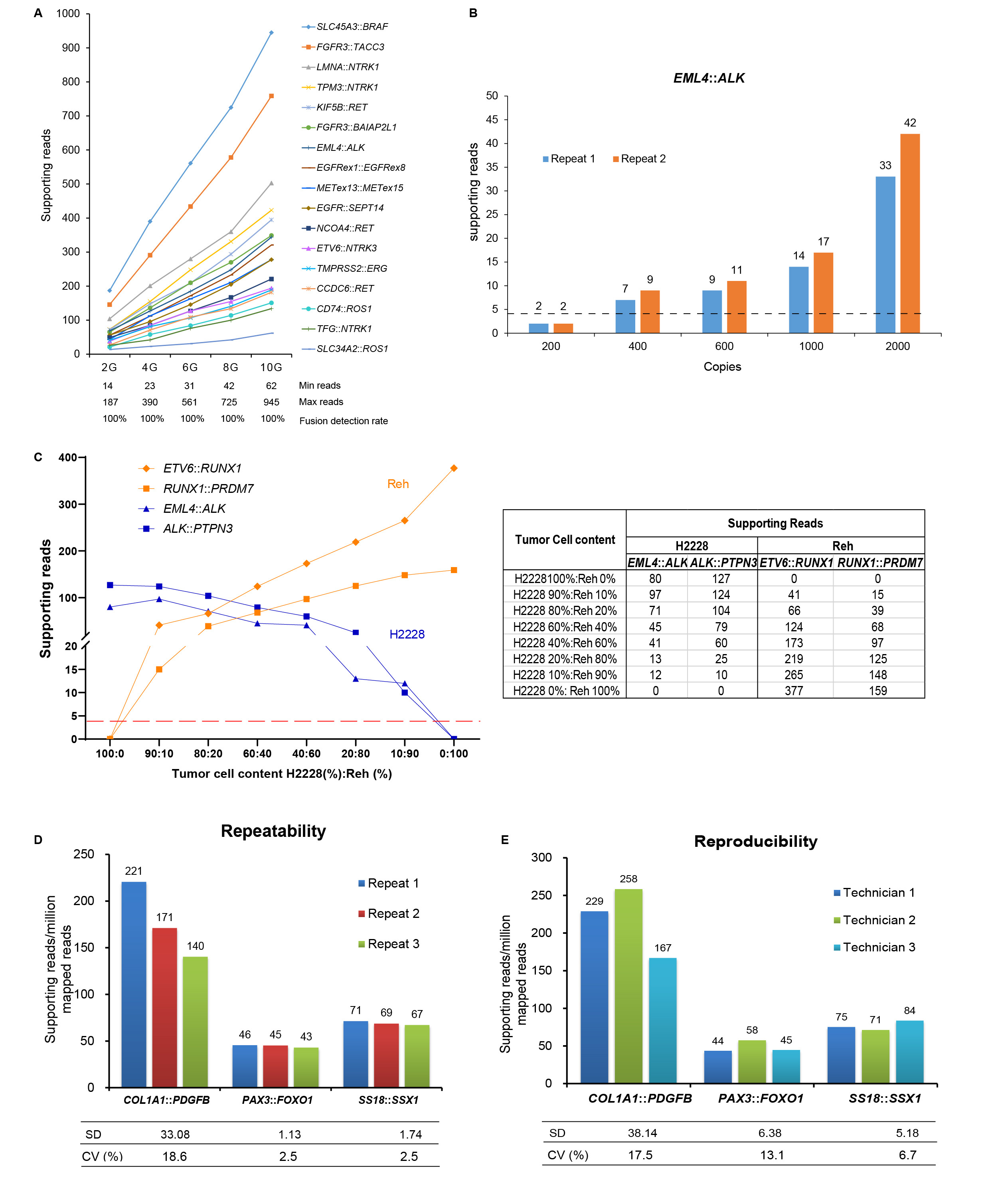


**Fig. S1 Analytical performance of RNA-based Fusioncapture panel.** **(A)** Fusion detection performance of Fusioncapture panel using reference material Seraseq® FFPE Tumor Fusion RNA v4 under different data sizes. The x-axis and y-axis represent the data size and fusion supporting reads, respectively. Each line represents one individual fusion. Each point indicates the supporting reads under a specific data size. **(B)** Detection limit of Fusioncapture panel using a serial dilution assay. The black dotted line represents the threshold of positive fusion. **(C)** Fusion detection for different tumor contents. The x-axis and y-axis represent the gradient mixture proportion of cell line Reh and H2228 and the number of fusion supporting reads, respectively. Each point indicates the mean supporting reads of three replicates under specific tumor content which was presented in the table on the right, and the red dotted line represents the threshold of positive fusion. **(D)** and **(E)** Intra-run repeatability and inter-run reproducibility of Fusioncapture panel for fusion detection using fusion positive clinical samples.


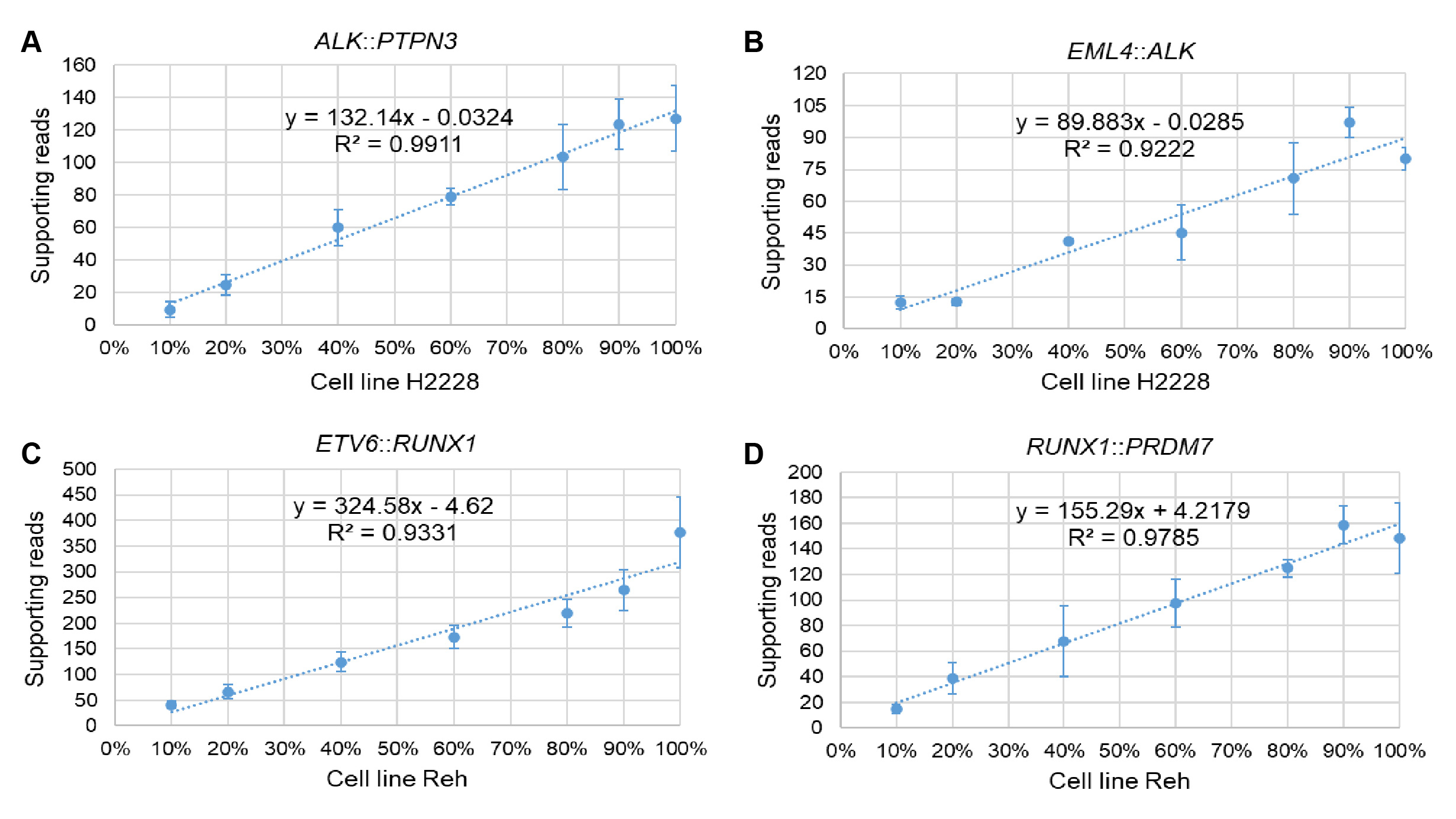


**Fig. S2 Fusion detection of the Fusioncapture panel in two cell lines H2228 and Reh under different tumor contents.** Scatterplot of supporting reads versus tumor content for *ALK*::*PTPN3* **(A)**, *EML4*::*ALK* **(B)**, *ETV6*::*RUNX1* **(C)**, and *RUNX1*::*PRDM7* fusion **(D)**. The x-axis and y-axis represent the cell content of target cell line and the number of fusion supporting reads, respectively. Each point indicates mean supporting reads of three replicates under specific tumor contents, and the bar represents the standard deviation.


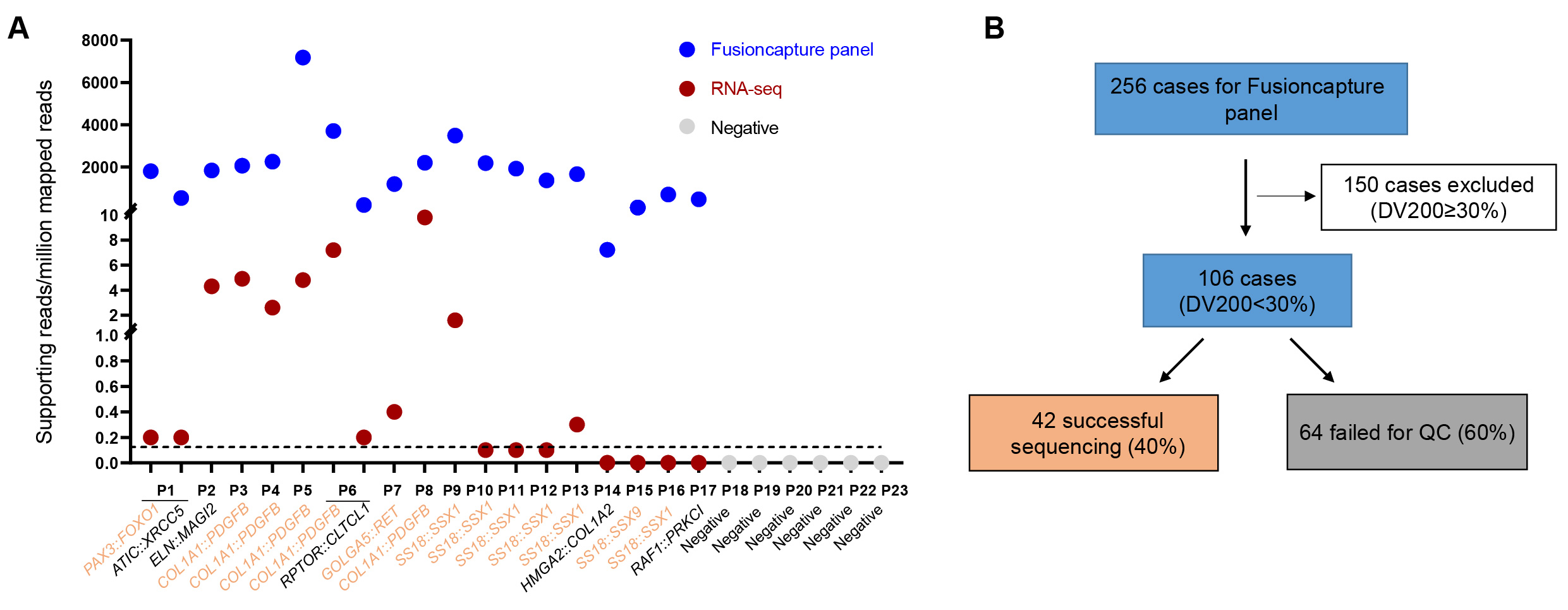


**Fig. S3 Comparison the performance of RNA-based Fusioncapture and RNA-seq in fusion detection.** **(A)** Comparison of Fusioncapture assay and RNA-seq in 23 FISH pre-validated clinical samples. The dotted line represents the normalized cutoff value (0.14). **(B)** The performance of Fusioncapture in low-quality clinical samples unacceptable for RNA-seq.


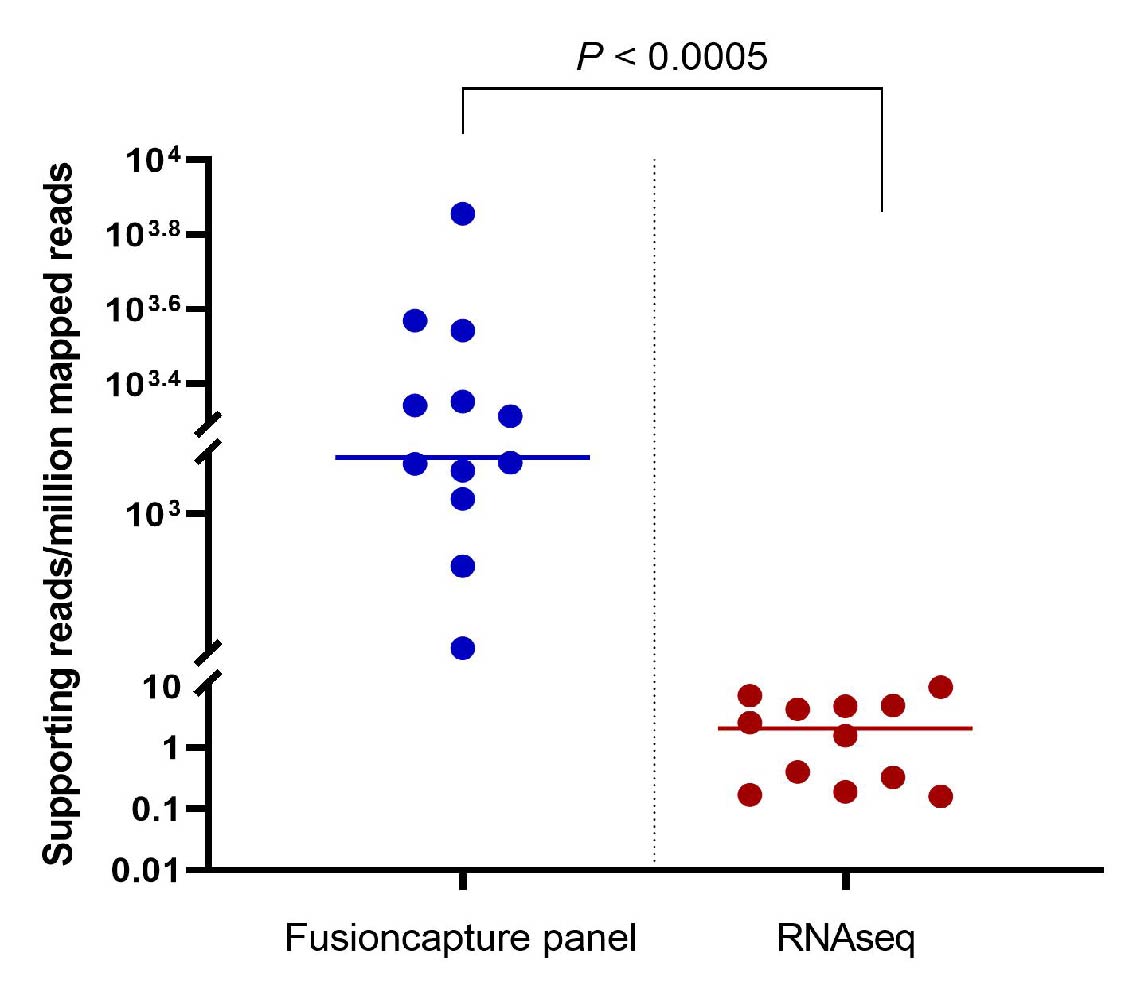


**Fig. S4 Scatter diagram of the number of fusion supporting reads detected by the RNA-based Fusioncapture panel and RNA-seq.** The supporting reads of 12 positive fusions identified by both methods were analyzed. Wilcoxon matched-pairs signed rank test was performed for the Fusioncapture panel and RNA-seq groups. The lines represent the median.


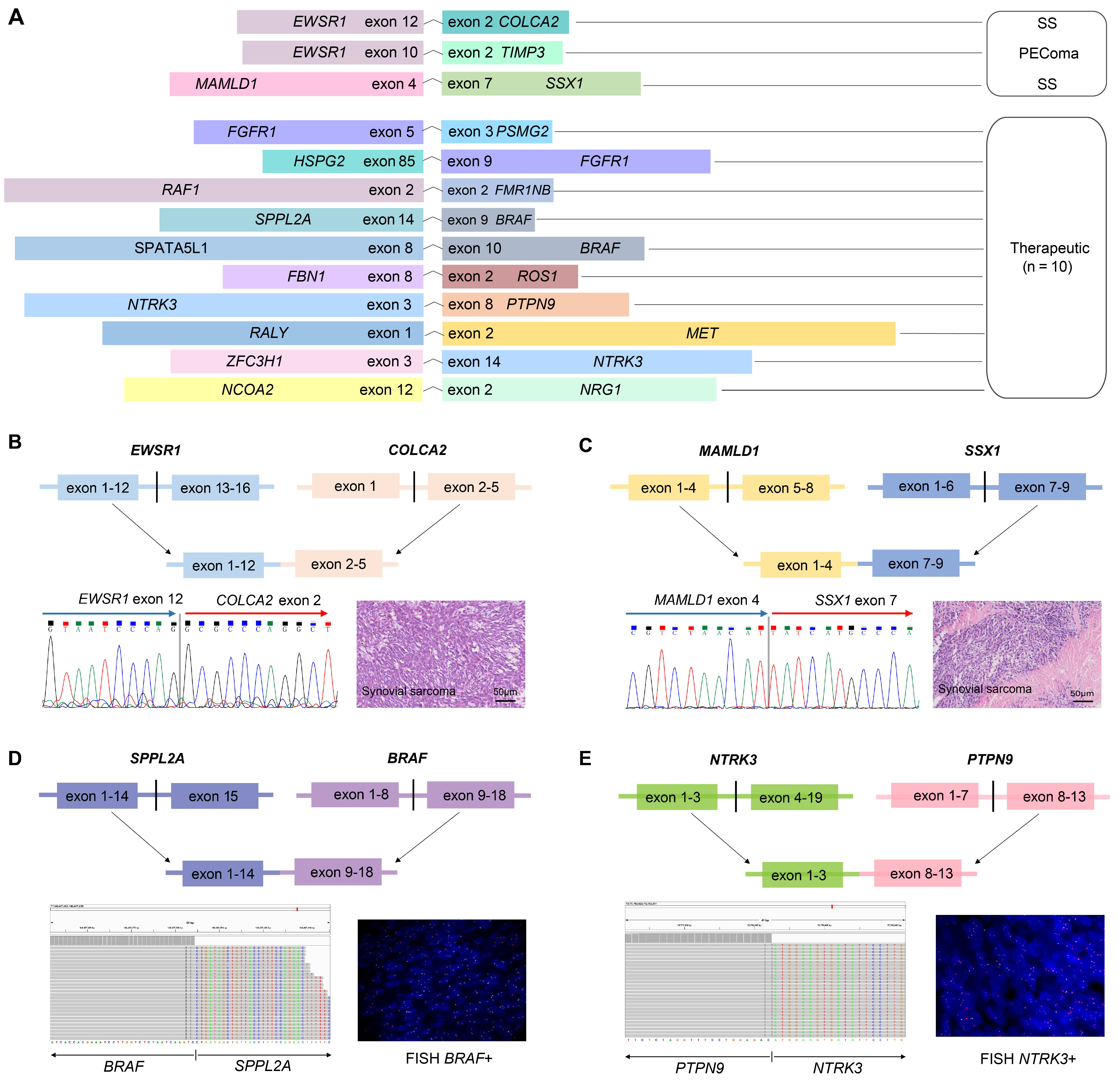


**Fig. S5 Novel fusion events found through Fusioncapture panel.** **(A)** Classification of the novel fusions according to their potential diagnostic or therapeutic utility. **(B)** and **(C)** Schematic diagram of *EWSR1*::*COLCA2* **(B)** and *MAMLD1*::*SSX1* **(C)** fusion with potential diagnostic value and the corresponding result of sanger sequencing and Hematoxylin and eosin staining of FFPE was shown. **(D)** and **(E)** Schematic diagram of *SPPL2A*::*BRAF* **(D)** and *NTRK3*::*PTPN9* **(E)** fusion with potential therapeutic value. Positive *BRAF* and *NTRK3* pattern with break-apart signals detected by FISH. PEComa, Perivascular epithelioid cell tumor; SS, Synovial sarcoma.
